# Overcoming the ties of gravity that bind us to Earth: Constantin von Economo, neurologist and pioneering aviator

**DOI:** 10.1055/s-0046-1829049

**Published:** 2026-10-06

**Authors:** Jéssica Yumi Lago Matsuda, Liria Matsuzawa Figueiredo, Allan Henrique Panisson, Matheus Kahakura Franco Pedro

**Affiliations:** 1 Faculdades Pequeno Príncipe, Medicine Program, Curitiba PR, Brazil.; 2 Instituto de Neurologia de Curitiba, Departamento de Neurologia, Curitiba PR, Brazil.

**Keywords:** Neurology, Parkinson Disease, Postencephalitic, History of Neurology, Aviation, World War I

## Abstract

Constantin von Economo was a brilliant neuroscientist whose 33 years of intense productivity are documented in over 4,200 pages of published works. He was best recognized for his notable research on encephalitis lethargica and the detailed cytoarchitectonic mapping of the human cerebral cortex. However, less widely known is that von Economo was also an aviation enthusiast, a skilled balloon and airplane pilot, who held prominent positions in aviation organizations, actively promoted international aeronautical meetings, and gained distinction as a pilot during World War I. Therefore, we aimed to revisit this remarkable but often overlooked aspect of his biography, emphasizing his love of the aeronautical arts and its impact on his medical career.

## INTRODUCTION

Constantin von Economo (1876–1931) was a brilliant neurologist who left a notable legacy in the intersections between neuroscience, neurology and psychiatry. 1 2 However, beyond his remarkable scientific achievements, von Economo was eminent in his age as a polymath and a daredevil of sorts, not only as a scientist. 2 3 A passionate and pioneering aviation enthusiast, he gained recognition for his accomplishments and contributions to the advancement of early aviation. 3 In this paper, the authors aim to shed light on this rather unknown facet of his life and the relationship between such diverse interest and his neurological legacy.

## BIOGRAPHICAL SKETCH

Born in Brăila, in Romania's region of Wallachia, von Economo belonged to a noble Greek lineage whose ancestry could be traced back to Macedonia. 1 He grew up in a privileged, sophisticated environment that nurtured him within the finest intellectual and cultural apparatus of the age. 1 2 In 1893, he moved to Vienna to begin studies in mechanical engineering, fulfilling his father's aspirations. 1 However, he soon became disillusioned with the curriculum and decided to shift his academic path; in 1895, he joined the School of Medicine of the University of Vienna, successfully graduating in 1901. 1 3 While still a student, von Economo marked the *début* of his scientific career by writing his first paper, an histological investigation on pituitary embryonic development in pigeons and chicks, which was published in 1899. 4 5 After graduation, he continued his studies in neuroscience by engaging in internships in several European centers. 1 2 In 1911, back in Vienna, he defended his thesis; 2 years later, he was appointed Lecturer in Psychiatry and Neurology at the University of Vienna and Professor in 1921 ( Figure 1 ). 1 2 3 4 5

**Figure 1:**
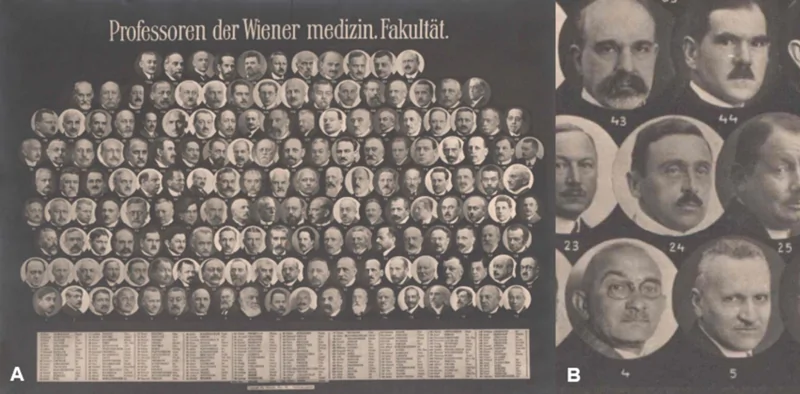
Source: *Österreichische Nationalbibliothek,* available at: https://digital.onb.ac.at/rep/osd/?11171BD9 ( **A** ) Group portrait of the professors of the Vienna Medical Faculty, 1926; ( **B** ) Constantin von Economo, identified as number 24 in the original image.

In 1917, von Economo presented to the Viennese Society for Psychiatry and Neurology his seminal findings on the raging epidemic of encephalitis lethargica, providing thorough descriptions of the neuropathological basis and full clinical spectrum of the disease, which occurred worldwide between 1915 and 1927 and represented a major cause of post-encephalitic parkinsonism; 6 7 his work with the disease garnered him widespread fame.

In 1925, von Economo and Georg Koskinas (1885–1975) created another veritable masterpiece that would cement his lasting reputation: their monumental Atlas and Textbook of Cytoarchitectonics of the Adult Human Cerebral Cortex. 4 8 Composed of 810 pages, compiled over the preceding 13 years, they histologically defined 107 cortical areas in a significantly more detailed depiction than Korbinian Brodmann's (1868–1918) famous monograph from 1909. 4 7 8

His brilliant life met an untimely death in 1931, at 55 years old, following a myocardial infarction complicated by a stroke 2 ; von Economo now rests in his family's mausoleum at *Santa Anna Cemetery* in Trieste. 3 5

## VON ECONOMO, THE AVIATOR

In 1907, von Economo first became an enthusiastic aeronaut. 9 At the time, although there were still no aeroplanes in Austria, there were balloons, inflated with hydrogen or liquefied petroleum gas; 9 10 Von Economo named his own balloon ‘Sonia’ 10 . In 1909, the Parisian newspaper *Le Matin* ( Figure 2A ) described von Economo's 13-hour trip from Paris to Linz, where he was honored with a visit from Archduke Joseph Ferdinand (1872–1942) 12 13 ( Figure 2B ).

**Figure 2:**
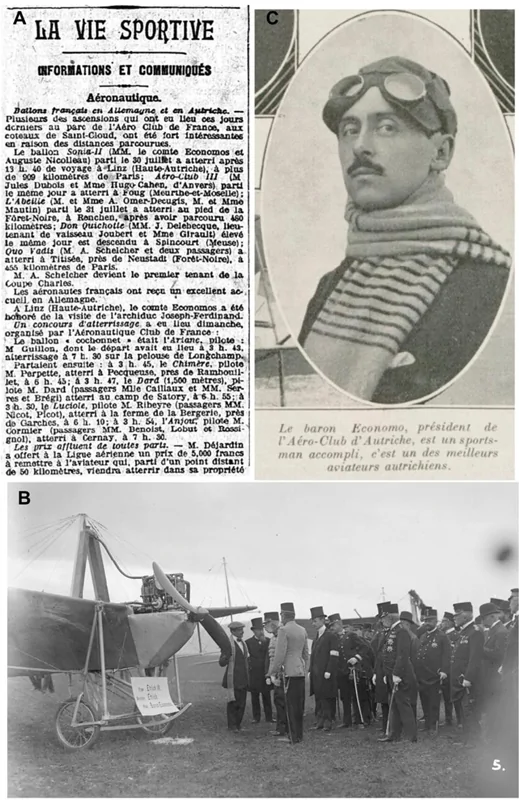
Source: *Bibliothèque Nationale de France, a* vailable at: https://gallica.bnf.fr/ark:/12148/bpt6k96053211/f14.item . ( **A** ) 1909 issue of the Parisian newspaper *Le Matin* reporting von Economo's 13-hour balloon trip from Paris to Linz (source: *Bibliothèque nationale de France, a* vailable at: https://gallica.bnf.fr/ark:/12148/bpt6k569143r/f4.item ); ( **B** ) Archduke Joseph Ferdinand visiting von Economo at the airfield in Wiener Neustadt, 1910 (source: *Österreichische Nationalbibliothek,* available at: https://digital.onb.ac.at/rep/osd/?10FCA9CF ); ( **C** ) Issue of the French magazine *La Vie au Grand Air,* in 1912, documenting Economo's achievement of traveling by balloon from Paris to Aspern.

In 1912, another French periodical, *La Vie au Grand Air,* documented the accomplishment of von Economo's long-sought ambition of traveling with his balloon from Paris to Aspern, a suburb of Vienna 14 ( Figure 2C ). The beginning of his active engagement in fixed-wing aviation started in 1908, when Economo purchased a *Voisin* airplane. 9 In 1910, he was elected President of the Austrian Aero-Club, a position he would hold for the next 16 years. 1 10 In 1912, he became the first Austrian citizen to obtain an international pilot license. That same year, he was awarded Field Pilot's Certificate No. 1 by the Austro-Hungarian military aviation authorities, in recognition of his instruction services. 5 10 11

## THE WARTIME AVIATOR

When the First World War broke out, Von Economo was the first registered reconnaissance pilot of the Austro-Hungarian forces. 4 Though he initially volunteered to serve as a pilot, he was instead first kept far from the front and was commissioned to train young pilots of the newly formed Air Force in Vienna. 10 In 1916, he served in the Russian front with the Second Automobile Corps, and later in the Lavis Squadron in the South Tyrol front. 4 10

He returned to the Vienna General Hospital as a military physician, where he treated soldiers with head injuries, an experience that eventually marked the beginning of his investigations into encephalitis lethargica. 5 For his contributions to the advancement of Austrian aeronautics, he was decorated with the Iron Cross. 5 In this way, it is worth noting that the same factor that granted him freedom to pursue his interest in aviation—the wealth of a noble family—allowed him to freely develop as a scholar, switching his graduation course on a whim at a time when few had such opportunities; furthermore, his position as a military physician, earned partly due to his pioneering aviation skills, landed him in the right place and the right time to lead his seminal research into encephalitis lethargica.

In 1927, marking the conclusion of his presidency of the Austrian Aero-Club, von Economo addressed his successor with words that resonate today: 9 10 “The millennia-old envy of man at the flight of birds and the drift of clouds has found form. [...] Creation impressed part of its creative craft upon the human brain, enabling us [...] to achieve increasingly higher possibilities along the way of our ascending evolution. And so, it is this same creative force of Nature, which in the course of eons gave the eagle its flight, that in recent decades enabled us humans to construct wings and overcome the ties of gravity that bind us to Earth.”
